# International consensus on the diagnosis and management of dumping syndrome

**DOI:** 10.1038/s41574-020-0357-5

**Published:** 2020-05-26

**Authors:** Emidio Scarpellini, Joris Arts, George Karamanolis, Anna Laurenius, Walter Siquini, Hidekazu Suzuki, Andrew Ukleja, Andre Van Beek, Tim Vanuytsel, Serhat Bor, Eugene Ceppa, Carlo Di Lorenzo, Marloes Emous, Heinz Hammer, Per Hellström, Martine Laville, Lars Lundell, Ad Masclee, Patrick Ritz, Jan Tack

**Affiliations:** 1 Translational Research Center for Gastrointestinal Disorders (TARGID), Department of Chronic Diseases, Metabolism and Ageing (ChroMetA), Catholic University of Leuven, Leuven, Belgium; 2 Gastroenterology Division, St Lucas Hospital, Bruges, Belgium; 3 2nd Department of Internal Medicine — Propaedeutic, Hepatogastroenterology Unit, Attikon University Hospital, Medical School, Athens University, Athens, Greece; 4 Department of Gastrosurgical Research and Education, Sahlgrenska Academy, University of Gothenburg, Gothenburg, Sweden; 5 Politechnic University of Marche, “Madonna del Soccorso” General Hospital, San Benedetto del Tronto, Italy; 6 Department of Gastroenterology and Hepatology, Tokai University School of Medicine, Isehara, Japan; 7 Division of Gastroenterology, Harvard Medical School, Beth Israel Deaconess Medical Center, Boston, MA USA; 8 Department of Endocrinology, University of Groningen, University Medical Center Groningen, Groningen, Netherlands; 9 Division of Gastroenterology, Ege University School of Medicine, Izmir, Turkey; 10 Department of Surgery, Indiana University School of Medicine, Indianapolis, IN USA; 11 Division of Pediatric Gastroenterology, Nationwide Children’s Hospital, Columbus, OH USA; 12 Department of Bariatric and Metabolic Surgery, Medical Center Leeuwarden, Leeuwarden, Netherlands; 13 Division of Gastroenterology and Hepatology, Department of Internal Medicine, Medical University of Graz, Graz, Austria; 14 Department of Medical Sciences, Gastroenterology/Hepatology, Uppsala University, Uppsala, Sweden; 15 Department of Endocrinology, Claude Bernard University, Lyon, France; 16 Department of Surgery Hospital, Karolinska University Hospital, Huddinge, Stockholm, Sweden; 17 Department of Gastroenterology-Hepatology, University Hospital Leiden, Leiden, Netherlands; 18 INSERM, U1027, 31073 Toulouse, France

**Keywords:** Obesity, Bariatric surgery, Multihormonal system disorders

## Abstract

Dumping syndrome is a common but underdiagnosed complication of gastric and oesophageal surgery. We initiated a Delphi consensus process with international multidisciplinary experts. We defined the scope, proposed statements and searched electronic databases to survey the literature. Eighteen experts participated in the literature summary and voting process evaluating 62 statements. We evaluated the quality of evidence using grading of recommendations assessment, development and evaluation (GRADE) criteria. Consensus (defined as >80% agreement) was reached for 33 of 62 statements, including the definition and symptom profile of dumping syndrome and its effect on quality of life. The panel agreed on the pathophysiological relevance of rapid passage of nutrients to the small bowel, on the role of decreased gastric volume capacity and release of glucagon-like peptide 1. Symptom recognition is crucial, and the modified oral glucose tolerance test, but not gastric emptying testing, is useful for diagnosis. An increase in haematocrit >3% or in pulse rate >10 bpm 30 min after the start of the glucose intake are diagnostic of early dumping syndrome, and a nadir hypoglycaemia level <50 mg/dl is diagnostic of late dumping syndrome. Dietary adjustment is the agreed first treatment step; acarbose is effective for late dumping syndrome symptoms and somatostatin analogues are preferred for patients who do not respond to diet adjustments and acarbose.

## Abstract (web summary)

Dumping syndrome is a frequent complication of oesophageal and gastric surgery, as well as bariatric surgery; however, guidance on how to manage patients with this condition is lacking. In this Evidence-based guideline, the authors use a Delphi consensus process to develop uniform guidance for the definition, diagnosis and management of dumping syndrome.

## Introduction

Dumping syndrome is a frequent complication of cancer and non-cancer oesophageal and gastric surgery, as well as bariatric surgery (also known as metabolic surgery). These interventions change gastric anatomy and innervation, which can enable a considerable amount of undigested food to reach the small intestine too rapidly^1–4^. Dumping syndrome comprises a constellation of symptoms that can be subdivided into early and late dumping syndrome symptoms, which can occur jointly or separately^1–8^. Typically, symptoms of early dumping syndrome occur within the first hour after a meal and include gastrointestinal symptoms (abdominal pain, bloating, borborygmi, nausea and diarrhoea) and vasomotor symptoms (flushing, palpitations, perspiration, tachycardia, hypotension, fatigue, desire to lie down and, rarely, syncope)^1,2^. The underlying mechanisms might involve osmotic effects, peptide hormone release and autonomic neural responses^1^. Symptoms of late dumping syndrome usually occur between 1 and 3 h after a meal and are primarily the manifestations of hypoglycaemia, which mainly results from an incretin-driven hyperinsulinaemic response after carbohydrate ingestion. Hypoglycaemia-related symptoms are attributable to neuroglycopenia (which is indicated by fatigue, weakness, confusion, hunger and syncope) and to vagal and sympathetic activation (indicated by perspiration, palpitations, tremor and irritability)^1,2^. The literature has referred to late dumping syndrome as ‘reactive hypoglycaemia’ or, after bariatric surgery, as ‘postbariatric hypoglycaemia’. However, on the basis of a common pathophysiology of rapid exposure of the small intestine to nutrients, which is also seen in early dumping syndrome (see subsequent discussion), we refer to this phenomenon as ‘late dumping syndrome’.

The prevalence of dumping syndrome depends on the type and extent of surgery, and on the criteria used to diagnose dumping syndrome. Dumping syndrome occurs in approximately 20% of patients undergoing vagotomy with pyloroplasty, in up to 40% of patients after Roux-en-Y gastric bypass (RYGB) or sleeve gastrectomy and in up to 50% of patients undergoing oesophagectomy^5,6,9–11^. Furthermore, dumping syndrome might also occur after Nissen fundoplication^12,13^. According to reports published in the past 15 years, bariatric surgery has become the main cause of postoperative dumping syndrome^14,15^. Dumping syndrome has mainly been reported after RYGB and partial gastrectomy^12,13^, but might also occur after restrictive bariatric procedures such as sleeve gastrectomy, vertical banded gastroplasty and the laparoscopic adjustable gastric band, which all reduce the volume capacity of the proximal stomach^4^. The rapid expansion in the use of bariatric interventions has therefore led to an increasing number of patients with dumping syndrome^16^.

Symptoms of dumping syndrome are often debilitating and emotionally distressing, they are associated with a substantial reduction in quality of life and might lead to considerable weight loss as a result of the patient avoiding food intake^17^. In spite of its effects, guidance is lacking on how to diagnose this condition, which is probably under-recognized. Moreover, established efficacious treatment options and management guidelines are lacking in the literature. Therefore, we used a Delphi consensus^1,9,10,17,18^ process to develop uniform guidance about the definition, diagnosis and management of dumping syndrome.

## Methods

The process was coordinated by a chair (J.T.) and a co-chair (E.S.), referred to as the chairs. The principal steps in the process were, first, selection of a Consensus Group consisting of international experts in dumping syndrome management with different clinical and scientific backgrounds. Second, draft statements were developed by the chairs and were refined by the Consensus Group after a preliminary voting round with feedback on the statements. Third, each expert was assigned to contribute to literature reviews on several topics to summarize the evidence to support each statement. Fourth, two rounds of online voting of the statements (and voting discussion) were undertaken until a stable level of consensus was reached. Fifth, grading of the strength and quality of the evidence and of the strength of the recommendations using grading of recommendations, assessment, development and evaluation (GRADE) criteria was conducted^19^. Agreement levels were determined as follows: A+, agree strongly; A, agree with minor reservation; A−, agree with major reservation; D−, disagree with major reservation; D, disagree with minor reservation; D+, disagree strongly.

For the Consensus Group, 18 multidisciplinary international experts (gastroenterologists, internists, nutritionists, surgeons and endocrinologists) from ten countries (Austria, Belgium, France, Greece, Italy, Japan, Netherlands, Sweden, Turkey and USA) were selected based on their participation in clinical trials and publications on dumping syndrome.

A literature research was conducted using a number of relevant keywords (medical subject headings (MeSH): dumping syndrome, hypoglycaemia and bariatric surgery). The chairs reviewed the list of publications and the relevant ones were stored in PDF format on a central server to which all Delphi panel members had access. The references cited in this paper are only a selection of the reviewed articles, chosen to clarify the discussion.

The chairs developed the initial 66 statements that were presented to the Consensus Group, who subsequently revised, expanded and consolidated the statements, ultimately providing 62 statements for the Delphi process^19^. The experts were then allocated to groups of three and each member also functioned as lead expert for one statement, generating a short summary of the available evidence for this statement using the papers on the central server as a literature source, which was further updated as needed. The statements covered the following aspects: definition, pathophysiology, diagnosis and treatment. Statements were revised by the chairs based on the feedback from the Consensus Group before the start of the first voting round and based on additional literature reviews, and also after each voting round.

Two voting rounds followed where each statement was presented with the evidence summary, and then the entire panel indicated the degree of agreement for the statement using a six-point Likert scale (Table 1). When at least 80% of the Consensus Group agreed (A+ or A) with a statement, this was defined as a consensus. All votes were fully anonymous, with no one knowing how anyone else voted.

**Table 1:** GRADE system^18^

Code	Quality of evidence	Definition
A	High	Further research is very unlikely to change our confidence in the estimate of effect; the statement can be supported by several high-quality studies with consistent results or in special cases by one large, high-quality multicentre trial
B	Moderate	Further research is likely to have an important effect on our confidence in the estimate of effect and might change the estimate; the statement can be supported by one high-quality study or several studies with some limitations
C	Low	Further research is very likely to have an important effect on our confidence in the estimate of effect and is likely to change the estimate; the statement can be supported by one or more studies with severe limitations
D	Very low	Any estimate of effect is very uncertain; the statement can be supported by expert opinion or one or more studies with very severe limitations, or there might be no direct research evidence

Six-point Likert scale for assessment of agreement level: A+, agree strongly; A, agree with minor reservation; A−, agree with major reservation; D−, disagree with major reservation; D, disagree with minor reservation; D+, disagree strongly. GRADE, grading of recommendations assessment, development and evaluation.

## Definition and symptoms

### 1: Dumping syndrome is a frequent complication of oesophageal, gastric or bariatric surgery

- Statement endorsed.
- Overall agreement 80%: A+ 65%, A 15%, A− 10%, D− 5%, D 0%, D+ 5%.
- Grade B.

### 2: Dumping syndrome consists of a constellation of symptoms that can be categorized as early dumping syndrome or late dumping syndrome

- Statement endorsed.
- Overall agreement 95%: A+ 90%, A 5%, A− 5%, D− 0%, D 0%, D+ 0%.
- Grade B.

Dumping syndrome occurs in approximately 20% of patients who undergo vagotomy with pyloroplasty, in up to 40% of patients after gastrectomy and in up to 50% of patients who undergo oesophagectomy^1,20^. Dumping syndrome has also been reported after Nissen fundoplication in both children and adults^13,20,21^. Over the past decade, bariatric surgery has become the principal cause of postoperative dumping syndrome. Furthermore, dumping syndrome has mainly been reported after RYGB and partial gastrectomy^7,20^. Moreover, among 450 patients who had undergone RYGB or sleeve gastrectomy, approximately one-third (34.2%) had postoperative symptoms consistent with postprandial hypoglycaemia, indicating the presence of late dumping syndrome^7,22^.

Dumping syndrome consists of a constellation of symptoms that can be subdivided into early dumping syndrome or late dumping syndrome on the basis of the time at which the symptoms appear and the presumed underlying pathophysiology^1,17,20^. Early dumping syndrome is characterized by gastrointestinal symptoms such as abdominal pain, bloating, borborygmi, nausea and diarrhoea, and vasomotor symptoms such as fatigue, desire to lie down after meals, flushing, palpitations, perspiration, tachycardia, hypotension and, rarely, syncope. Symptoms of late dumping syndrome are related to neuroglycopenia (indicated by fatigue, weakness, confusion, hunger and syncope) and autonomic and/or adrenergic reactivity (indicated by perspiration, palpitations, tremor and irritability).

### 3: Early dumping syndrome symptoms occur within the first hour after a meal

- Statement endorsed.
- Overall agreement 95%: A+ 80%, A 15%, A− 5%, D− 0%, D 0%, D+ 0%.
- Grade B.

### 4: Early dumping syndrome is characterized by gastrointestinal symptoms (such as abdominal pain, cramps, bloating, borborygmi, nausea and diarrhoea) and vasomotor symptoms (such as fatigue, desire to lie down after meals, flushing, palpitations, perspiration, tachycardia, hypotension and, rarely, syncope)

- Statement endorsed.
- Overall agreement 100%: A+ 95%, A 5%, A− 0%, D− 0%, D 0%, D+ 0%.
- Grade B.

### 5: Late dumping syndrome usually occurs 1–3 h after a meal and is characterized by (reactive) hypoglycaemia

- Statement endorsed.
- Overall agreement 95%: A+ 85, A 10%, A− 5%, D− 0%, D 0%, D+ 0%.
- Grade A.

Symptoms of early dumping syndrome are attributed to rapid passage of nutrients to the small intestine, which activates a cascade of pathophysiological events. The arrival of hyperosmolar contents in the small intestine triggers a shift of fluid from the intravascular component to the intestinal lumen, leading to decreased circulating blood volume, duodenal or jejunal distention and release of several gastrointestinal peptide hormones. These changes trigger symptoms of early dumping syndrome such as tachycardia, hypotension and, rarely, syncope as well as abdominal cramps. Provocative tests for assessing dumping syndrome (a modified oral glucose tolerance test (OGTT)) have shown that most of these symptoms, or their consequences (increased pulse rate and rise in the haematocrit level), are already present at 30 min after the meal^1,17,20^. Late dumping syndrome can be attributed to the development of hyperinsulinaemic, or reactive, hypoglycaemia. Rapid delivery of carbohydrates to the small intestine leads to high glucose concentrations, which triggers a hyperinsulinaemic response, and subsequent hypoglycaemia^1,20^. After bariatric surgery, it takes 3 months to 1 year for clinical signs of hypoglycaemia to appear, perhaps because insulin sensitivity increases as weight loss occurs^23^.

### 6: Early dumping syndrome is the typical and most frequent manifestation of dumping syndrome and might occur in isolation or in association with symptoms of late dumping syndrome

- Statement endorsed.
- Overall agreement 90%: A+ 50%, A 40%, A− 5%, D− 5%, D 0%, D+ 0%.
- Grade B.

The literature is not clear on the relative prevalence of early dumping syndrome versus late dumping syndrome. However, studies involving glucose tolerance testing show a very high occurrence of increased pulse rate, a marker of early dumping syndrome, and a lower occurrence of hypoglycaemia, a marker of late dumping syndrome. These findings suggest that early dumping syndrome might be more prevalent than late dumping syndrome^1,17,20^. Using the Mine score for symptom assessments, a higher proportion of patients reported early dumping syndrome than late dumping syndrome after gastric surgery for cancer^24^. By contrast, isolated late dumping syndrome (hypoglycaemia as the only symptom) might affect up to 25% of patients who have undergone surgery for gastric cancer^5,7^.

### 7: In severe cases, dumping syndrome is associated with a substantial reduction in quality of life

- Statement endorsed.
- Overall agreement 95%: A+ 85%, A 10%, A− 5%, D− 0%, D 0%, D+ 0%.
- Grade B.

Several studies in the 1990s reported the effect of dumping syndrome symptoms after gastrectomy on daily functioning^25–27^. More recent studies, using the Gastrointestinal Quality of Life Index (GIQLI), the Short-Form 36 or the RAND-36 questionnaire showed values well below the healthy population range^17,27,28^. A specific postgastrectomy quality of life instrument was developed, which documented considerably impaired quality of life after gastrectomy, with an important negative effect of dumping syndrome symptoms^23^. However, this scale uses the presence and number of symptoms of both early and late dumping syndrome and, hence, is driven by the physician rather than added to by the patient. Specific patient-administered quality of life scales for dumping syndrome are lacking.

### 8: In severe cases, dumping syndrome is associated with weight loss

- Statement endorsed.
- Overall agreement 95%: A+ 65%, A 30%, A− 0%, D− 0%, D 5%, D+ 0%.
- Grade B.

It is well established that bariatric surgery is associated with weight loss and a risk of dumping syndrome. However, literature from the peptic ulcer surgery era and data from gastric and oesophageal cancer surgery around the turn of the century also show that dumping syndrome might lead to weight loss^29,30^. In addition, procedures that aim to prevent dumping syndrome such as vagus sparing oesophagectomy or pyloric reconstruction with gastric cancer surgery are associated with less weight loss than traditional resection procedures^19,31–35^. Weight loss was also reported in adults and children with dumping syndrome after Nissen fundoplication^13,21,36^.

## Pathophysiology

### 9: In early dumping syndrome, probably due to hyperosmolality of the food, rapid fluid shifts occur from the plasma compartment to the intestinal lumen. The fluid shift accounts for part of the cardiovascular early dumping response

- Statement endorsed.
- Overall agreement 85%: A+ 45%, A 40%, A− 5%, D− 5%, D 5%, D+ 0%.
- Grade B.

### 10: Dumping syndrome might occur after gastric surgery with removal of the barrier function of the pylorus, resulting in the rapid delivery of a substantial amount of undigested solid food to the small intestine

- Statement endorsed.
- Overall agreement 100%: A+ 75%, A 25%, A− 0%, D− 0%, D 0%, D+ 0%.
- Grade B.

### 11: Dumping syndrome might occur after gastric surgery that reduces gastric volume capacity, resulting in the rapid delivery of a substantial amount of undigested solid food to the small intestine

- Statement endorsed.
- Overall agreement 90%: A+ 70%, A 20%, A− 0%, D− 5%, D 5%, D+ 0%.
- Grade B.

### 12: Movement of fluid into the small bowel related to dumping syndrome might cause distention and contribute to cramp-like contractions, bloating and diarrhoea

- Statement endorsed.
- Overall agreement 90%: A+ 55%, A 35%, A− 10%, D− 0%, D 0%, D+ 0%.
- Grade B.

### 13: In early dumping syndrome, release of several gastrointestinal hormones, including vasoactive agents, incretins and glucose modulators, induces gastrointestinal symptoms and haemodynamic effects

- Statement endorsed.
- Overall agreement 95%: A+ 60%, A 35%, A− 5%, D− 0%, D 0%, D+ 0%.
- Grade B.

Gastric surgery can reduce gastric volume or remove the barrier function of the pylorus, which allows rapid delivery of food into the small intestine. Hyperosmolar small bowel content causes a shift of fluid from the vascular compartment to the intestinal lumen, resulting in a reduced circulating volume of plasma, tachycardia, hypotension and, rarely, syncope. Movement of fluid into the small bowel might also cause duodenal or jejunal distension and generate abdominal symptoms such as cramping, diarrhoea, pain and bloating. The fluid shift is confirmed by the rise in haematocrit level at 30 min during the glucose tolerance test that is seen in a subset of the patients studied^1,17,20^. A compensatory drop in atrial natriuretic peptide secretion occurs^37^. However, volume shifts are probably not the only mechanism as intravenous fluid substitution was found to be ineffective in preventing early dumping syndrome symptoms^38^. The role of gastric volume is illustrated by the occurrence of dumping syndrome after the sleeve gastrectomy bariatric procedure and also after a Nissen fundoplication^5,6,13,21,36^.

The second mechanism involved in the pathophysiology of early dumping syndrome is probably enhanced release of several gastrointestinal hormones, including vasoactive agents (such as neurotensin and vasoactive intestinal peptide), incretins (such as glucagon-like peptide 1 (GLP1)), YY gastric inhibitory polypeptide and glucose modulators (such as insulin and glucagon)^1,17,20,39–47^. The levels of all gut peptides do not rise in dumping syndrome; for instance, levels of substance P and motilin did not increase^42,43^. In addition to chemosensing, duodenal or jejunal distention might also contribute to the release of these gastrointestinal hormones. Anecdotal evidence in support of this observation includes the frequent finding of a dilated small bowel during barium radiography in patients with dumping syndrome (interpreted in part as hypersecretion) and the induction of symptoms by duodenal distention in healthy volunteers^48–50^. Through their actions, the released peptide hormones might contribute to both the gastrointestinal and the cardiovascular effects of dumping syndrome.

### 14: Rapid delivery of undigested carbohydrates to the small intestine might result in high concentrations of glucose that induce a hyperinsulinaemic response, resulting in subsequent hypoglycaemia and related late dumping syndrome

- Statement endorsed.
- Overall agreement 100%: A+ 80%, A 20%, A− 0%, D− 0%, D 0%, D+ 0%.
- Grade A.

### 15: An exaggerated GLP1 response is the key mediator of the hyperinsulinaemic and hypoglycaemic effect that is characteristic of late dumping syndrome

- Statement endorsed.
- Overall agreement 90%: A+ 50%, A 40%, A− 5%, D− 0%, D 0%, D+ 5%.
- Grade B.

Enteral glucose administration induces enhanced insulin release relative to intravenous administration, which is the so-called incretin effect. Glucose-dependent insulinotropic polypeptide (also called gastric inhibitory polypeptide) and GLP1 have a pivotal role in the incretin effect. Patients with reactive hypoglycaemia after gastric surgery have an increased GLP1 response, and the rising levels of GLP1 are correlated with insulin release^1,20,41,51–53^. In addition, infusion of the GLP1 receptor antagonist exendin (9–39) amide was able to correct reactive hypoglycaemia after gastric bypass surgery^52^. These observations support an exaggerated GLP1 response as a key mediator of the hyperinsulinaemic and reactive hypoglycaemic effect in late dumping syndrome.

### 16: Dumping syndrome occurring after bariatric surgery can be associated with weight loss

- Statement endorsed.
- Overall agreement 80%: A+ 60%, A 20%, A− 10%, D− 5%, D 5%, D+ 0%.
- Grade B.

### 17: Dumping syndrome occurring after bariatric surgery can contribute to weight loss

- Statement endorsed.
- Overall agreement 80%: A+ 60%, A 20%, A− 10%, D− 10%, D 0%, D+ 0%.
- Grade B.

The success of RYGB surgery is usually attributed to gastric volume reduction and calorie malabsorption secondary to the bypass of the small intestine, which leads to markedly changed eating behaviour and meal patterns^54,55^. Other mechanisms that contribute to postoperative weight loss include reduced hunger, increased satiation, increased energy expenditure and altered taste perception, all of which might be mediated by alterations in gastrointestinal and central neuroendocrine signalling^56–59^. It has been proposed that dumping syndrome, through its adverse effects on food tolerance and intake, might be an essential component of the weight reduction after bariatric surgery^55^. However, no trial has demonstrated that participants who have dumping syndrome symptoms lose more weight than those who do not have dumping syndrome, and this finding was confirmed in dedicated studies^11,60^. Hence, dumping syndrome occurring after bariatric surgery is not a desired effect and must be considered a procedure complication as it can impair quality of life and digestive functions. Furthermore, to what extent a low frequency or mild intensity manifestation of dumping syndrome is a normal event after bariatric surgery also has not been established.

### 18: Reactive hypoglycaemia in patients who did not undergo upper gastrointestinal surgery can be a manifestation of idiopathic dumping syndrome

- Statement not endorsed.
- Overall agreement 75%: A+ 20%, A 55%, A− 10%, D− 15%, D 0%, D+ 0%.
- Grade C.

The literature has reported on small numbers of patients with reactive hypoglycaemia without prior surgery who exhibit late hypoglycaemia in an oral glucose challenge test^61^. Rapid gastric emptying seemed to be the common underlying mechanism^62^. As a result of associated gastrointestinal symptoms, the rapid gastric emptying and the frequent association with postprandial diarrhoea, reactive hypoglycaemia seems to be an underlying mechanism that is similar to that of dumping syndrome after surgery. These patients responded well to dietary adjustment (frequent small meals)^61^.

## Symptom-based diagnosis

### 19: Dumping syndrome should be suspected based on the concurrent presentation of multiple suggestive symptoms in patients who have undergone gastric or oesophageal surgery

- Statement endorsed.
- Overall agreement 100%: A+ 70%, A 30%, A− 0%, D− 0%, D 0%, D+ 0%.
- Grade B.

While this statement seems obvious, in clinical practice dumping syndrome is insufficiently known and is often missed. Furthermore, diagnosis and the appropriate treatment are often delayed for several months or years. Profound fatigue after meal ingestion, with the need to lie down, in patients with an appropriate surgical history is an important clinical clue^1,20^. In patients with suspected dumping syndrome, the diagnosis can be established using symptom-based questionnaires, by oral glucose challenge testing and other diagnostic investigations (see subsequent sections).

Mechanical obstruction or subobstruction (narrowing of the lumen that slows but does not block passage of content) needs to be considered in the differential diagnosis of gastrointestinal symptoms in patients with a surgical history suggestive of dumping syndrome. However, mechanical alterations after surgery might mimic some of the early symptoms of dumping syndrome, but will not be associated with hypoglycaemia and will not result in any of the abnormal features during a glucose tolerance test that are seen in patients with dumping syndrome^63,64^. Clinical judgement will guide the extent to which additional testing is done to evaluate the postsurgical anatomy.

### 20: Symptom-based questionnaires, such as the Sigstad’s score and the Arts dumping questionnaire, can be used to identify patients with clinically meaningful dumping syndrome symptoms

- Statement not endorsed.
- Overall agreement 70%: A+ 30%, A 40%, A− 15%, D− 10%, D 5%, D+ 0%.
- Grade C.

### 21: Sigstad’s dumping score questionnaire is sensitive to therapy

- Statement not endorsed.
- Overall agreement 30%: A+ 20%, A 10%, A− 35%, D− 15%, D 5%, D+ 15%.
- Grade C.

### 22: The diagnostic accuracy of the Sigstad’s scoring questionnaire is similar in patients undergoing peptic ulcer surgery, bariatric surgery or upper gastrointestinal cancer surgery

- Statement not endorsed.
- Overall agreement 20%: A+ 20%, A 20%, A− 20%, D− 20%, D 10%, D+ 10%.
- Grade C.

### 23: The diagnostic accuracy of the Sigstad’s scoring questionnaire is acceptable for identifying early dumping syndrome after peptic ulcer surgery only

- Statement not endorsed.
- Overall agreement 45%: A+ 0%, A 45%, A− 10%, D− 25%, D 15%, D+ 5%.
- Grade C.

### 24: Arts dumping-severity score questionnaire is able to discriminate patients with early and late dumping syndrome

- Statement not endorsed.
- Overall agreement 75%: A+ 50%, A 25%, A− 10%, D− 10%, D 5%, D+ 0%.
- Grade B.

### 25: Arts dumping-severity score questionnaire is sensitive to therapeutic effects

- Statement not endorsed.
- Overall agreement 65%: A+ 25%, A 40%, A− 15%, D− 15%, D 5%, D+ 0%.
- Grade B.

### 26: The Dumping Symptom Rating Scale patient self-assessment questionnaire is accurate in determining symptom severity and frequency in dumping syndrome both preoperatively and postoperatively (for example, RYGB)

- Statement not endorsed.
- Overall agreement 45%: A+ 20%, A 25%, A− 30%, D− 15%, D 10%, D+ 0%.
- Grade C.

The Sigstad’s scoring system, which was proposed in 1970, is designed to aid the diagnosis of dumping syndrome by allocating points to symptoms, which are then added up^3^ (Box 1). The total points are summarized into a calculated diagnostic index; if the score is above 7 it is suggestive of dumping syndrome and if the score is <4 then other diagnoses need to be considered^65^. Scores of 5 and 6 represent a grey area as what they mean for the diagnosis of dumping syndrome is unclear. However, these values and their interpretation were established for patients undergoing peptic ulcer surgery and the diagnostic accuracy of the Sigstad’s scoring questionnaire in patients undergoing bariatric surgery or upper gastrointestinal cancer surgery has not been established. A study of 50 patients who underwent gastric bypass found a Sigstad’s score indicative of dumping syndrome in 42% of the patients, without correlation to the amount of weight loss^11^. However, a study in 24 patients without type 2 diabetes mellitus who were undergoing laparoscopic sleeve gastrectomy showed a disconnect between the score and hypoglycaemia during the glucose challenge test^11^.

The Sigstad’s scoring system was mainly proposed as a diagnostic aid. To our knowledge, its sensitivity to treatment interventions has not been studied. Laurenius and colleagues reported a modified use of the Sigstad’s score, at 15-min intervals during an OGTT, and the area under the curve distinguished patients with or without symptoms of dumping syndrome after RYGB^66^. Whether this method was superior to the classic score was not reported; in addition, no diagnostic cut-offs were identified. Taking these findings together, the Sigstad’s score correctly identifies a substantial group of patients with dumping syndrome after all types of surgeries; however, there are insufficient data to compare the diagnostic efficacy of Sigstad’s scoring system after bariatric surgery versus cancer or peptic ulcer surgery.

In the dumping severity score developed by Arts and colleagues, symptoms of early and late dumping syndrome (which have eight and six symptoms, respectively) are scored on a four-point Likert scale^17^ (Box 2). Severity scores are obtained by adding the individual scores of the symptoms displayed by the patient. This score was mainly used as an index of severity. The diagnostic performance has not been addressed. In addition, no threshold was established for any of the subscores (late or early). Hence, although this score quantifies symptoms, its discriminatory value for early versus late dumping syndrome has not been addressed.

In open-label studies of the somatostatin analogues octreotide and lanreotide, early, late and total Arts scores were altered following treatment^17,67^. By contrast, no statistically significant improvement in Arts scores was found in a pilot study with the somatostatin analogue pasireotide^68^; however, the authors argued that the applied dose might have been too high, inducing gastrointestinal adverse effects.

A report published in 2010 describes the use of a visual analogue scale (severity of symptoms indicated on a 10-cm line with severity ranging left to right from 0 to intolerable) survey to evaluate seven symptoms of early dumping syndrome and six symptoms of late dumping syndrome in more than 1,000 patients who had undergone gastrectomy for gastric cancer^24^. The analysis generated a very low cut-off for diagnosing dumping syndrome (visual analogue scale score >10 mm on a single item on the questionnaire), but surprisingly a higher cut-off (45 mm) gave similar diagnostic yield^24^. No other reports on the use of this questionnaire are available to date.

The Dumping Symptom Rating Scale is a questionnaire based on input from a multidisciplinary team of physicians^8^. This scale comprises nine symptoms addressing early dumping syndrome, one on symptoms related to drinking fluids and one related to consuming sweetened drinks. A summary score is generated by multiplying individual scores for severity (range 1–9) and frequency (range 1–8) for each item and adding these up. Content validity, internal consistency and construct validity were established in a large patient cohort. Test–retest reliability was less consistent. Variable correlations were found with items from the Gastrointestinal Symptom Rating Scale. Furthermore, responsiveness to therapy has not been assessed for this scale.

Box 1 Dumping syndrome symptoms according to the Sigstad’s scoring system The Sigstad’s scoring system was developed in the era of peptic ulcer surgery and assigns points to each of 16 symptoms of dumping syndrome, and the total points are used to calculate a diagnostic index. A diagnostic index >7 is suggestive of dumping syndrome, whereas a score <4 suggests that other diagnoses should be considered.

- Shock +5
- Fainting (syncope), unconsciousness +4
- Desire to lie or sit down +4
- Breathlessness (dyspnoea) +3
- Weakness, exhaustion +3
- Sleepiness, drowsiness, apathy, falling asleep +3
- Palpitation +3
- Restlessness +2
- Dizziness +2
- Headaches +1
- Feeling of warmth, sweating, pallor, clammy skin +1
- Nausea +1
- Abdominal fullness, meteorism +1
- Borborygmus +1
- Eructation −1
- Vomiting −4

Box 2 Dumping syndrome symptoms according to the Arts scoring system In the Arts scoring system, a distinction is made between eight symptoms of early dumping syndrome (first hour after the meal) and six symptoms of late dumping syndrome (occurring after the first hour). Each symptom is scored for severity on a 0–3 Likert scale (absent to severe). Early and late dumping syndrome scores are calculated as, respectively, the sum of the eight symptoms of early dumping syndrome and the six symptoms of late dumping syndrome. The total severity score for dumping syndrome is the sum of severities of all symptoms.

Early dumping syndrome symptoms

- Sweating
- Flushing
- Dizziness
- Palpitations
- Abdominal pain
- Diarrhoea
- Bloating
- Nausea

Late dumping syndrome symptoms

- Sweating
- Palpitations
- Hunger
- Drowsiness and/or unconsciousness
- Tremor
- Irritability

Severity score

For each symptom: 0 = absent, 1 = mild, 2 = relevant and 3 = severe

## Diagnostic testing

### 27: Spontaneous plasma levels of glucose <2.8 mmol/l (50 mg/dl) are indicative of late dumping syndrome

- Statement endorsed.
- Overall agreement 80%: A+ 50%, A 30%, A− 0%, D− 5%, D 15%, D+ 0%.
- Grade B.

### 28: Spontaneous plasma levels of glucose <3.3 mmol/l (60 mg/dl) are indicative of late dumping syndrome

- Statement not endorsed.
- Overall agreement 45%: A+ 30%, A 15%, A− 30%, D− 10%, D 10%, D+ 5%.
- Grade C.

Hypoglycaemia is relevant when it is accompanied by symptoms that are relieved by ingestion of carbohydrates. This is referred to as Whipple’s triad in the literature on insulinoma and postbariatric surgery hypoglycaemia^69,70^. Although no definitive cut-off values were defined for random glucose concentrations in a literature review published in 2015, the use of glucose concentrations below 3.3 mmol/l has been suggested as a sensitive cut-off value for meal-induced hypoglycaemia during OGTT or a mixed meal tolerance test^71^. This cut-off value has also been used by other authors in paediatric patients^72^ as well as in adult patients^73^. This cut-off value is used after a glucose load, with the patient sitting in a chair over a long period of time for repeated blood samples and hence using very little energy. In that respect it seems acceptable to require a stricter cut-off, such as 2.8 mmol/l rather than 3.3 mmol/l, for spontaneous events. In a study using continuous glucose monitoring on reactive hypoglycaemia, the cut-off for hypoglycaemia events was 70 mg/dl (3.9 mmol/l)^74^. In another study measuring continuous glycaemia in patients with reactive hypoglycaemia, a cut-off of 3.3 mmol/l (60 mg/dl) was used, but only 5% of symptom episodes were below this threshold^75^. In a study with acarbose, a threshold of 60 mg/dl was also used to assess hypoglycaemia^76^.

Data from the literature on diabetes mellitus treated with insulin show an increase in the occurrence of symptomatic hypoglycaemia from levels of 3.9 mmol/l and below. From an extensive database analysis, the majority of hypoglycaemia episodes above 3.5 mmol/l remained asymptomatic, and it was concluded that values between 3.5 mmol/l and 4.0 mmol/l are probably of minor importance, and that a cut-off of 3.4 mmol/l (54 mg/dl) is appropriate in the setting of patients with diabetes mellitus treated with insulin^77^. Ambulatory glycaemia monitoring studies used lower cut-off levels for hypoglycaemia (3.1 mmol/l and even 2.2 mmol/l), but no consensus is currently reached on thresholds^78^. The 2.2 mmol/l threshold was proposed as this level of hypoglycaemia leads to notable and sustained cognitive impairment (from the UK Hypoglycaemia Study Group)^79^.

In summary, no consensus on the glucose concentration that defines hypoglycaemia is available from the literature (either literature on dumping syndrome or the broader literature). A cut-off of 3.3 mmol/l seems reasonable, but 2.8 mmol/l is more predictably associated with symptoms, and was supported by the current Delphi consensus.

### 29: Continuous glucose monitoring is beneficial in dumping syndrome

- Statement not endorsed.
- Overall agreement 65%: A+ 35%, A 30%, A− 15%, D− 5%, D 15%, D+ 0%.
- Grade C.

### 30: Continuous glucose monitoring is beneficial for identifying complex cases of dumping syndrome

- Statement not endorsed.
- Overall agreement 70%: A+ 50%, A 20%, A− 10%, D− 15%, D 5%, D+ 0%.
- Grade C.

### 31: Continuous glucose monitoring is a reproducible assay in identifying dumping syndrome

- Statement not endorsed.
- Overall agreement 35%: A+ 10%, A 25%, A− 35%, D− 5%, D 15%, D+ 10%.
- Grade C.

Anecdotal reports suggest that monitoring glycaemia is useful in patients with suspected dumping syndrome^80–84^. One case report and one therapeutic study used continuous glucose monitoring as an outcome variable to assess the effect of acarbose and dietary measures^75,82^. However, the diagnostic accuracy of continuous glucose monitoring has not been compared with that of dumping provocative tests or diagnostic questionnaires or been evaluated as a marker of therapeutic outcome.

### 32: Sigstad’s scoring system can identify early dumping syndrome by diagnosing signs and symptoms such as a high pulse rate or increased haematocrit level that are indicative of hypovolaemia during an OGTT

- Statement not endorsed.
- Overall agreement 60%: A+ 25%, A 35%, A− 15%, D− 10%, D 10%, D+ 5%.
- Grade B.

In its original report, the Sigstad’s diagnostic questionnaire test was proposed to be combined with an OGTT in the diagnostic work-up and the Sigstad’s scoring system; the scores and outcomes of the provocative test differed in 25 patients with or without dumping syndrome after gastrectomy^64^. The Sigstad’s scoring system primarily aims to identify early dumping syndrome through signs and symptoms such as a high pulse rate or increased haematocrit level that are indicative of hypovolaemia. The index does not use hypoglycaemia (a marker of late dumping syndrome) and hence it is likely to underestimate prevalence and severity of dumping syndrome.

### 33: In the modified glucose tolerance test, patients with suspected dumping syndrome ingest 75 g of glucose in solution after an overnight fast; blood concentrations of glucose, haematocrit level, pulse rate and blood pressure are measured before and at 30-min intervals up to 180 min after ingestion

- Statement endorsed.
- Overall agreement 95%: A+ 60%, A 35%, A− 0%, D− 0%, D 5%, D+ 0%.
- Grade B.

### 34: The modified OGTT is considered positive for early dumping syndrome based on the presence of an early (30 min) increase in haematocrit level >3% or an increase in pulse rate >10 bpm 30 min after ingestion

- Statement endorsed.
- Overall agreement 90%: A+ 70%, A 20%, A− 0%, D− 10%, D 0%, D+ 0%.
- Grade B.

### 35: The modified OGTT is considered positive for late dumping syndrome based on the development of late (60–180 min after ingestion) hypoglycaemia (<50 mg/dl)

- Statement endorsed.
- Overall agreement 80%: A+ 40%, A 40%, A− 15%, D− 5%, D 0%, D+ 0%.
- Grade B.

### 36: The modified OGTT is considered positive for late dumping syndrome based on the development of late (60–180 min after ingestion) hypoglycaemia (<60 mg/dl)

- Statement not endorsed.
- Overall agreement 60%: A+ 40%, A 20%, A− 20%, D− 10%, D 5%, D+ 5%.
- Grade B.

### 37: The modified OGTT is an assay for identifying dumping syndrome that has good reproducibility

- Statement not endorsed.
- Overall agreement 35%: A+ 10%, A 25%, A− 35%, D− 15%, D 15%, D+ 0%.
- Grade C.

### 38: The modified OGTT has a good specificity but a low sensitivity for dumping syndrome

- Statement not endorsed.
- Overall agreement 45%: A+ 10%, A 35%, A− 55%, D− 0%, D 0%, D+ 0%.
- Grade B.

### 39: To increase the low sensitivity of the modified OGTT, especially in patients after gastric bypass surgery, a validated questionnaire should be added to the test

- Statement not endorsed.
- Overall agreement 55%: A+ 20%, A 35%, A− 30%, D− 0%, D 15%, D+ 0%.
- Grade B.

### 40: The mixed meal tolerance test is more sensitive than the modified OGTT for diagnosing late dumping syndrome

- Statement not endorsed.
- Overall agreement 35%: A+ 10%, A 25%, A− 35%, D− 10%, D 15%, D+ 5%.
- Grade C.

The OGTT is now the preferred diagnostic test for dumping syndrome^1,20,85^. This test generally involves the ingestion of 50 g or 75 g glucose in solution, but glucose doses between 25 g and 100 g have been used by various authors^69^. Blood concentration of glucose, haematocrit level, pulse rate and blood pressure are measured at 30-min intervals for up to 3 h after the ingestion.

The test is considered positive if late (120–180 min) hypoglycaemia occurs, or if an early (30 min) increase in haematocrit level of more than 3% occurs. The most sensitive sign of early dumping syndrome seems to be a rise in the pulse rate by more than 10 bpm after 30 min^1,17,20,67,68,85,86^. Most studies have considered glycaemia below 60 mg/dl, usually occurring between 90 min and 180 min after ingestion, as diagnostic of late dumping syndrome. Hypoglycaemia is a marker of late dumping syndrome and hence, if present, allows a diagnosis of dumping syndrome. Its absence does not exclude a diagnosis of dumping syndrome as early dumping syndrome might be present in the absence of late dumping syndrome^1^. In the literature from the past few years, this level of glycaemia mostly occurred at 120 min, 150 min or 180 min^1,17,67,68,85,86^. No systematic analysis has compared cut-offs of 50 mg/dl versus 60 mg/dl. However, based on our experience and supported by the available literature^1,17,67,68,85,86^, a cut-off of 50 mg/dl during an OGTT, during which no physical activity is performed, might underestimate the prevalence of late dumping syndrome and decrease diagnostic sensitivity. Nevertheless, the current consensus selected 50 mg/dl as a cut-off value for defining late hypoglycaemia in dumping syndrome. OGTTs have also been used for long-term follow-up studies^6^. The clinical unit where the test is performed should be familiar with symptomatic hypoglycaemia during the OGTT and how to manage it. However, the literature does not report problematic adverse effects during repeated OGTT in patients with dumping syndrome^17,66–68,85,86^.

The reproducibility of the modified OGTT has not been studied separately. However, data from a phase II clinical trial involving pasireotide in patients with dumping syndrome, where progressively increasing doses of pasireotide were added with repeated OGTT testing, show that up to 50% of patients continued to display hypoglycaemia during treatment^86^. This observation supports the concept that the test might be reproducible for the occurrence of hypoglycaemia. In these studies, at OGTT testing, the persistence of a rise in pulse rate or a rise in haematocrit level at 30 min with treatment was lower than the persistence of hypoglycaemia, suggesting that either this aspect is less reproducible, or that pasireotide is more effective in treating early dumping syndrome (pulse rate rise and haematocrit rise) than in treating late dumping syndrome (hypoglycaemia).

The OGTT might demonstrate hypoglycaemia after gastric bypass surgery even in the absence of symptoms suggestive of late dumping syndrome^69^. The diagnostic accuracy of this test is therefore likely to be low. When patients without diabetes mellitus were tested before and after a bariatric procedure, predominantly gastric bypass, half of all patients developed hypoglycaemia; however, none had hypoglycaemic symptoms, which suggests the test has low specificity^87^. For this reason, it has been suggested that measured hypoglycaemia in patients after bariatric surgery is only relevant when associated with symptoms that are relieved by ingestion of carbohydrates (Whipple’s triad)^69^. On the basis of these considerations, the guideline for evaluation and management of adult hypoglycaemic disorders from the Endocrine Society that was published in 2009 rejected the use of the OGTT for testing postprandial hypoglycaemia, but this guideline did not mention or consider dumping syndrome^88^. Whether the accuracy or sensitivity of the OGTT for dumping syndrome can be improved by adding questionnaires (such as Sigstad’s, Arts or Mine questionnaires) has not been evaluated.

As an alternative, the mixed meal tolerance test has been recommended to confirm the diagnosis of symptomatic hypoglycaemia after gastric bypass^69^. In this test, patients with suspected dumping syndrome ingest a mixed meal containing carbohydrates, fat and proteins after an overnight fast, and blood samples are collected prior to the meal and at 30-min intervals for up to 2 h afterwards to measure glycaemic and insulin profiles. Compared to the OGTT, only a limited number of studies have reported on the mixed meal test^53,89^. No head-to-head comparisons are available, and hence evaluating respective sensitivities for the tests cannot be done reliably. However, this test was claimed to have a lower rate of hypoglycaemia occurrence than OGTT^53,71,89^.

### 41: A gastric emptying test showing rapid emptying rate can be used to confirm a diagnosis of dumping syndrome

- Statement not endorsed.
- Overall agreement 30%: A+ 10%, A 20%, A− 30%, D− 15%, D 20%, D+ 5%.
- Grade B.

### 42: Gastric emptying tests have low sensitivity and specificity for dumping syndrome

- Statement endorsed.
- Overall agreement 85%: A+ 35%, A 50%, A− 5%, D− 5%, D 5%, D+ 0%.
- Grade B.

Although rapid gastric emptying is a key mechanism in dumping syndrome, the diagnostic accuracy of rapid gastric emptying seems to be low. First, the test is not applicable after total gastrectomy. Second, rapid gastric emptying might occur in conditions other than dumping syndrome — for instance, functional dyspepsia^90,91^. Furthermore, initial rapid gastric emptying is enough to trigger symptoms of dumping syndrome, but these symptoms, including nausea, might in turn delay gastric emptying, such that the overall value of gastric emptying rate is within the normal range, as has been reported in a number of series^18,68^. On the basis of these limitations, gastric emptying testing seems to be of low utility in diagnosing dumping syndrome.

## Treatment

### 43: Dietary modification is the initial approach, and is usually beneficial for the majority of patients

- Statement endorsed.
- Overall agreement 100%: A+ 80%, A 20%, A− 0%, D− 0%, D 0%, D+ 0%.
- Grade B.

### 44: Clinicians should advise patients with dumping syndrome to reduce the amount of food consumed at each meal; moreover, patients should delay fluid intake until at least 30 min after meals

- Statement endorsed.
- Overall agreement 90%: A+ 55%, A 35%, A− 10%, D− 0%, D 0%, D+ 0%.
- Grade B.

### 45: Rapidly absorbable carbohydrates should be eliminated from the diet to prevent symptoms of late dumping syndrome, such as hypoglycaemia

- Statement endorsed.
- Overall agreement 90%: A+ 80%, A 10%, A− 10%, D− 0%, D 0%, D+ 0%.
- Grade B.

### 46: Patients with dumping syndrome should be advised to eat a diet consisting of foods high in fibre and rich in protein, eaten slowly and chewed well

- Statement endorsed.
- Overall agreement 80%: A+ 55%, A 25%, A− 15%, D− 5%, D 0%, D+ 0%.
- Grade C.

### 47: Patients with dumping syndrome should be advised to lie down for 30 min after meals to reduce the symptoms of hypovolaemia

- Statement not endorsed.
- Overall agreement 55%: A+ 35%, A 20%, A− 25%, D− 10%, D 5%, D+ 5%.
- Grade C.

Dietary modification is the initial treatment approach where patients are advised to reduce the amount of food ingested at each meal, to postpone fluid intake until at least 30 min after meals and to eliminate rapidly absorbable carbohydrates, which are present in all sweet foods and drinks, for instance. Instead, patients are advised to eat a diet consisting of foods that are high in fibre and rich in protein; consumption of fruit and vegetables is encouraged, whereas alcoholic beverages are better avoided^1^. Case series in adults and children argue in favour of the benefit of dietary intervention, but the focus of these studies is on late dumping syndrome (hypoglycaemia)^1,20,92–96^. Patients should also eat slowly and chew well^1^. Controlled data showing benefit from protein-rich foods or delaying fluid intake are not available in the literature. Education about the glycaemic index of different foods might also be helpful for patients with dumping syndrome. In addition, patients can be advised to lie down for 30 min after meals to delay gastric emptying and reduce the symptoms of hypovolaemia; however, evidence for this approach is lacking^8,96–99^.

### 48: Dietary supplements that increase the viscosity of food (such as guar gum, pectin and glucomannan) are a good second-line (after diet) treatment for symptoms of dumping syndrome

- Statement not endorsed.
- Overall agreement 40%: A+ 20%, A 20%, A− 45%, D− 5%, D 10%, D+ 0%.
- Grade B.

A number of studies have evaluated the use of supplements that increase food viscosity, such as guar gum, pectin and glucomannan, in patients with dumping syndrome^45,100–106^ (Table 2). The rationale is that the increased consistency of the meal will slow the release of nutrients to the small intestine. Several studies evaluated the ingestion of up to 15 g of guar gum or pectin with each meal to slow gastric emptying, reduce the release of gastrointestinal hormones, improve hyperglycaemia and control symptoms of dumping syndrome^45,100–106^. One study reported that glucomannan statistically significantly improved glucose tolerance but had no effect on glucose absorption in children with dumping syndrome^104^. However, the palatability and tolerability of such dietary supplements are usually poor^1^.

**Table 2:** Summary of studies evaluating pectin, guar gum and glucomannan in dumping syndrome

Study	*n*	Treatment	Result
Jenkins et al.^104^	9	Pectin 14.5 g, single administration prior to OGTT	Improved symptoms and glycaemia levels (normalized in 46%) during OGTT
Jenkins et al.^105^	11	Pectin 14.5 g, single administration prior to OGTT	Improved postprandial levels of glucose, insulin and enteroglucagon; reduced hypoglycaemia
Leeds et al.^153^	11	Pectin 15 g, single administration prior to OGTT	Improved vasomotor symptoms and glycaemia levels, lower insulin levels and slower gastric emptying during OGTT
Lawaetz et al.^44^	4	Pectin 15 g, single administration prior to OGTT	Reduced vasomotor symptoms, lower levels of insulin, glucagon, neurotensin and gastric inhibitory polypeptide, and slower initial gastric emptying during OGTT
Andersen et al.^100^	5	Pectin 5 g, single administration prior to muffin meal	No effect on symptoms or gastric emptying rate
Harju and Larmi^101^	11	Guar gum 5 g with meals	Improvement of symptoms
Harju et al.^102^	11	Guar gum 5 g with meals	Slowing of gastric emptying
Harju and Makela^103^	11	Guar gum 5 g with a glucose challenge meal	Improvement of symptoms and hyperglycaemia after a glucose challenge meal
Kneepkens et al.^106^	8 children	Glucomannan 1.3 g, single administration prior to OGTT	Improvement of glucose tolerance, no effect on glucose absorption; however, no consistent effect on symptoms was seen

OGTT, modified oral glucose tolerance test.

### 49: Pharmacological intervention has to be considered in the management of dumping syndrome in patients who do not respond to dietary modification

- Statement endorsed.
- Overall agreement 90%: A+ 80%, A 10%, A− 10%, D− 0%, D 0%, D+ 0%.
- Grade B.

### 50: Acarbose can be used as a treatment for symptoms of late dumping syndrome

- Statement endorsed.
- Overall agreement 85%: A+ 55%, A 30%, A− 10%, D− 5%, D 0%, D+ 0%.
- Grade B.

### 51: Acarbose does not affect symptoms of early dumping syndrome

- Statement not endorsed.
- Overall agreement 75%: A+ 35%, A 40%, A− 0%, D− 15%, D 5%, D+ 5%.
- Grade B.

In patients with dumping syndrome who are not responding to dietary interventions the use of pharmacological therapy needs to be considered because the efficacy of pharmacotherapy might be higher than dietary interventions and is better supported by mechanistic and controlled trials; furthermore, the effect of dumping syndrome on quality of life is considerable. Nevertheless, a conservative approach can be considered in patients who prefer not to progress to pharmacological therapy, provided that no major symptoms, such as hypoglycaemia leading to diminished awareness, coma or inability to drive or function, are present.

Acarbose is an alpha-glycosidase inhibitor that slows the release of monoglycerides from nutritional carbohydrates. The available studies with acarbose are summarized in Table 3. Most studies are fairly small and of short duration. They consistently show that acarbose improves glucose tolerance, reduces gastrointestinal hormone release and reduces the incidence of hypoglycaemia, which is the main feature of late dumping syndrome^12,76,107–116^. No specific evidence of an effect on symptoms of early dumping syndrome is available. However, the absence of a detailed distinction of symptoms of early and late dumping syndrome in a few studies means that it cannot definitely be excluded that acarbose can also have an effect on treating symptoms of early dumping syndrome. The usual dose of acarbose is 50–100 mg three times a day with meals. The main adverse effect is flatulence and related gastrointestinal symptoms such as bloating due to carbohydrate malabsorption. For adherence reasons, patients should be informed about this effect as an inevitable adverse effect due to the mechanism of action of the drug.

**Table 3:** Summary of studies evaluating acarbose in dumping syndrome

Study	*n*	Treatment	Result
McLoughlin et al.^111^	10	Acarbose 100 mg single administration prior to OGTT	Improved symptoms and hyperglycaemia and hypoglycaemia during OGTT; reduced rise in plasma levels of gastric inhibitory polypeptide and insulin; no change in gastric emptying rate
Gerard et al.^108^	24	Acarbose 100 mg single administration prior to OGTT	Improved hyperglycaemia and hypoglycaemia during OGTT; reduced rise in plasma levels of insulin; inhibition of glucose-induced suppression of glucagon
Lyons et al.^110^	13	Acarbose 50 mg single administration prior to standard breakfast	Significant attenuation of hyperglycaemia; reduced rise in plasma levels of gastric inhibitory polypeptide, enteroglucagon and insulin; no influence on plasma levels of vasoactive intestinal polypeptide and somatostatin; no significant effect on symptoms
Hasegawa et al.^109^	6	Acarbose 50–100 mg 3 times per day before meals for a month	Attenuation of glucose fluctuations and improvement of dumping syndrome symptoms (uncontrolled)
Ozgen et al.^113^	21	Acarbose 150 mg per day before meals for 2 weeks and 300 mg per day for the remainder of the 3-month treatment period	Reduced early hyperglycaemic and hyperinsulinaemic response; reduced reactive hypoglycaemia
Ng et al.^12^	6	Acarbose 12.5 mg before a meal	Improved postprandial hypoglycaemia
De Cunto et al.^115^	4	Acarbose 25–100 mg before meals	Stabilized postprandial levels of glucose
Valderas et al.^114^	8	Acarbose 100 mg before a meal	Avoided postprandial hypoglycaemia; reduced hyperinsulinaemic response; reduced GLP1 secretion
Ritz et al.^76^	8	Acarbose 50–100 mg, 3 times per day for 6 weeks	Eliminated dumping syndrome symptoms and improved CGM profile
Speth et al.^107^	9	Acarbose 50–100 mg, pectin 4.2 g, acarbose 50 mg plus pectin 4.2 g, placebo, after standard breakfast	Acarbose and acarbose plus pectin inhibited postprandial hyperglycaemia and hypoglycaemia; acarbose plus pectin inhibited hyperinsulinaemia; acarbose, pectin and combination therapy reduced hypoglycaemic symptoms

CGM, continuous glucose monitoring; GLP1, glucagon-like peptide 1; OGTT, modified oral glucose tolerance test.

### 52: Diazoxide, a potassium channel activator that inhibits calcium-induced insulin release, can be used as a treatment for symptoms of late dumping syndrome

- Statement not endorsed.
- Overall agreement 50%: A+ 25%, A 25%, A− 25%, D− 15%, D 10%, D+ 0%.
- Grade C.

Diazoxide inhibits insulin secretion by opening ATP-sensitive potassium channels in pancreatic β-cells, and is therefore expected to prevent the hypoglycaemia of late dumping syndrome. The use of diazoxide for symptoms of late dumping syndrome is only mentioned anecdotally in the literature, as case reports and case series^117–120^. Published in 2016, a multicentre, retrospective, systematic case series of six patients with hyperinsulinaemic hypoglycaemia after bariatric surgery reported that diazoxide reduced the number and severity of hypoglycaemic events in three patients^119^. In a small prospective case series, published as an abstract only, diazoxide significantly improved late hypoglycaemia without having any statistically significant effects on other parameters^120^.

### 53: Somatostatin analogues are the preferred treatment option for patients with well‑established dumping syndrome who do not respond to initial dietary modification with or without acarbose treatment

- Statement endorsed.
- Overall agreement 90%: A+ 65%, A 25%, A− 5%, D− 0%, D 5%, D+ 0%.
- Grade B.

### 54: Both short-acting and long-acting formulations of somatostatin analogues are efficacious for treating symptoms of both early and late dumping syndrome

- Statement not endorsed.
- Overall agreement 75%: A+ 40%, A 35%, A− 15%, D− 5%, D 5%, D+ 0%.
- Grade B.

### 55: Short-acting somatostatin analogue formulations are more effective than long-acting formulations at improving symptoms of dumping syndrome

- Statement endorsed.
- Overall agreement 80%: A+ 55%, A 25%, A− 20%, D− 0%, D 0%, D+ 0%.
- Grade B.

### 56: The need for repeated injections of somatostatin analogues throughout the day is a major limitation to the long-term administration of short-acting formulations

- Statement endorsed.
- Overall agreement 90%: A+ 60%, A 30%, A− 10%, D− 0%, D 0%, D+ 0%.
- Grade B.

Somatostatin analogues are able to slow the rate of gastric emptying, slow small bowel transit, inhibit the release of gastrointestinal hormones, inhibit insulin secretion and inhibit postprandial vasodilation; these analogues are therefore of potential benefit for both early and late dumping syndromes. The efficacy of somatostatin analogues for dumping syndrome was initially supported by case series^120–128^ and subsequently by several randomized controlled trials^2,28,67,129,130^ (Table 4). The evidence applies to symptoms of both early and late dumping syndromes, after peptic ulcer, bariatric and cancer surgeries. Studies from the Netherlands and Belgium have shown that both short-acting and long-acting somatostatin analogues provide symptomatic benefit, but patients prefer the long-acting preparations, probably because of the lower number of injections needed^17,28,67,121,126^. Data from glucose challenge tests and the assessment of haematocrit level and/or pulse rate provide objective evidence of the efficacy of octreotide and pasireotide in both early and late dumping syndrome^17,68^. Penning and colleagues have shown how long-acting formulations are more effective than short-acting formulations in increasing body weight and improving quality of life^28^. However, in a study by Arts and colleagues, better symptom control was obtained with short-acting octreotide three times a day than with long-acting octreotide administered monthly^17,28^.

**Table 4:** Summary of studies evaluating somatostatin analogues in dumping syndrome

Study	*n*	Treatment	Result
***Short-acting somatostatin analogues***			
Hopman et al.^122^	12	Octreotide 50 µg versus placebo prior to OGTT	Improved symptoms of dumping syndrome and suppression of postprandial rise in pulse rate; reduced peak insulin and higher nadir glycaemia; slowing of gastrointestinal transit
Primrose and Johnston^125^	10	Octreotide 50 µg versus 100 µg versus placebo prior to OGTT	Reduced symptoms of early dumping syndrome and abolished symptoms of late dumping syndrome; suppression of early dumping-associated changes in haematocrit and pulse rate; inhibition of hypoglycaemia
Tulassay et al.^127^	8	Octreotide 50 µg versus placebo prior to OGTT	Suppression of rise in pulse rate and haematocrit; suppression of rise in plasma levels of vasoactive intestinal polypeptide; inhibition of postprandial hypoglycaemia; inhibition of rise in plasma levels of insulin and gastric inhibitory polypeptide
Geer et al.^128^	10	Octreotide 100 µg versus placebo prior to a dumping provocative meal	Prevention of development of symptoms of dumping syndrome and diarrhoea; prevention of late hypoglycaemia and of the rise in plasma levels of glucose, glucagon, pancreatic polypeptide, neurotensin and insulin; delayed gastric emptying and intestinal transit
Richards et al.^129^	6	Octreotide 100 µg versus placebo prior to a dumping provocative meal	Prevention of symptoms of dumping syndrome; induction of migrating motor complex phase 3 in the small intestine; less postprandial intestinal motor activity
Gray et al.^130^	9	Octreotide 100 µg versus placebo prior to a dumping provocative meal	Suppression of rise in pulse rate; inhibition of insulin release; prevention of hypoglycaemia; inhibition of symptoms of dumping syndrome
Hasler et al.^131^	8	Octreotide 50 µg versus placebo prior to OGTT	Suppression of rise in pulse rate; inhibition of symptoms of dumping syndrome and diarrhoea; no influence on change in haematocrit; inhibition of insulin release; prevention of hypoglycaemia; no influence on gastric emptying rate
Arts et al.^17^	30	Octreotide 50 µg prior to OGTT	Suppression of rise in pulse rate and haematocrit; inhibition of postprandial hypoglycaemia; inhibition of rise in plasma levels of insulin; improvement of symptoms of early and late dumping syndrome
Deloose et al.^68^	9	Crossover placebo or pasireotide 300 µg for 2 weeks	Inhibition of postprandial hypoglycaemia; slowed gastric emptying rate
Tack et al.^86^	43	3-month dose-escalation study with pasireotide 50–200 µg (subcutaneous) followed by extension with monthly long-acting 10 mg or 20 mg injections	Improvement of symptoms of late and early dumping syndrome and signs on the OGTT
***Long-acting somatostatin analogues***			
Arts et al.^17^	30	Octreotide long-acting release 20 mg (intramuscular)	Suppression of rise in pulse rate and haematocrit; inhibition of postprandial hypoglycaemia; inhibition of rise in plasma levels of insulin; improvement of symptoms of early and late dumping syndrome and quality of life; preferred by patients over short-acting formulation
Wauters et al.^67^	24	Crossover study with placebo or lanreotide 90 mg (intramuscular)	Improvement of symptoms of early but not late dumping syndrome.

OGTT, modified oral glucose tolerance test.

Indeed, long-acting octreotide (intramuscularly) might offer the potential advantage of less frequent injections that are more convenient for the patient and might reduce injection aversion, which was often reported with short-acting octreotide injections (subcutaneously)^1,17^. Octreotide use might be associated with the occurrence of hypoglycaemia as an adverse effect. Hence, in theory, a worsening or different pattern of hypoglycaemia with octreotide is possible. However, this effect has not been reported in any of the octreotide studies for dumping syndrome to date, which suggests this is not a relevant issue in this population^17,28,67,68,121–131^. Paediatric usage of octreotide or other analogues is not yet supported by solid evidence as no specific studies on dumping syndrome in this population of patients are available^132^.

### 57: Constant enteral nutrition via a feeding jejunostomy can be effective for the management of refractory dumping syndrome

- Statement not endorsed.
- Overall agreement 55%: A+ 25%, A 30%, A− 15%, D− 10%, D 20%, D+ 0%.
- Grade C.

### 58: Continuous enteral feeding via a gastrostomy tube can be effective for the management of dumping syndrome after Nissen fundoplication

- Statement not endorsed.
- Overall agreement 40%: A+ 15%, A 25%, A− 20%, D− 20%, D 20%, D+ 0%.
- Grade C.

Evidence supporting statements 57 and 58 is scarce, and is mainly derived from a few case reports, including symptoms of dumping syndrome after Nissen fundoplication^1,133^. In a few case reports, insertion of a gastrostomy tube into the remnant stomach after RYGB reversed neuroglycopenic symptoms^70,134^. This treatment option can only be considered for severe refractory cases as it is invasive and as symptoms might improve with time (see subsequent section).

### 59: Conservative management approaches should be pursued before attempting surgical re-intervention as patients with dumping syndrome might experience symptomatic improvement over time

- Statement endorsed.
- Overall agreement 85%: A+ 65%, A 20%, A− 10%, D− 0%, D 5%, D+ 0%.
- Grade B.

### 60: Patients with severe hypoglycaemia after RYGB who do not respond adequately to dietary modification and pharmacologic intervention should be considered for surgical re-intervention

- Statement not endorsed.
- Overall agreement 70%: A+ 40%, A 30%, A− 25%, D− 5%, D 0%, D+ 0%.
- Grade C.

### 61: The association between hypoglycaemia after RYGB and nesidioblastosis that might result in serious and refractory neuroglycopenic symptoms might be resolved with pancreatic resection (distal, subtotal and total pancreatomies — distal pancreatectomy with or without splenectomy is the most common)

- Statement not endorsed.
- Overall agreement 45%: A+ 15%, A 30%, A− 30%, D− 20%, D 5%, D+ 0%.
- Grade C.

### 62: The proportion of patients with symptom resolution is generally higher for gastric bypass reversal or gastric pouch restriction than for pancreatic resection

- Statement not endorsed.
- Overall agreement 50%: A+ 15%, A 35%, A− 20%, D− 20%, D 5%, D+ 5%.
- Grade C.

No real data are available on the natural history of dumping syndrome, and hence whether patients improve over time has not been established. A number of studies have reported on surgical re-intervention in the treatment of severe hypoglycaemia in patients after RYGB using a variety of surgical techniques (such as bypass reversal, pouch restriction and interposed intestinal loops), with variable outcomes^135–145^. In one report, three patients with dumping syndrome and refractory hypoglycaemia had insufficient benefit of the reversal of their gastric bypass and they ultimately required partial pancreatectomy for control of neuroglycopenia^117^.

Indeed, pancreatic nesidioblastosis (a hyperplasia of islet cells that is potentially driven by elevated secretion of GLP1) has been implicated in the pathogenesis of refractory hypoglycaemia; on the basis of several reported cases, subtotal pancreatectomy is the suggested treatment^117,137,146–150^. Other studies, however, claim that hyperinsulinaemic hypoglycaemia after gastric bypass surgery is not accompanied by islet hyperplasia or increased β-cell turnover, and hence that nesidioblastosis is not established as the cause of late hypoglycaemia in these patients^151^. In fact, the available evidence supports a functional hyperinsulinism driven at least in part by high levels of glucose and incretin after meals rather than increased islet cell mass, thereby questioning the concept of nesidioblastosis^151,152^.

A meta-analysis of results from 14 studies that enrolled a total of 75 patients who underwent surgical interventions for severe hypoglycaemia after RYGB reported hypoglycaemia resolution in 67% of patients after pancreatic resection, 76% after gastric bypass reversal and 82% after pouch restriction^147^. However, a follow-up study by the Mayo Clinic group showed that 25% of patients not responding to other therapeutic measures also experienced no benefit from partial pancreatectomy and that, with time, recurrences of hypoglycaemia also occurred, further questioning the benefit of these interventions^152^. Thus, randomized controlled trials are needed to determine the true efficacy of gastric bypass reversal or gastric pouch versus subtotal or total pancreatic resection.

## Recommendations

**Fig. 1:**
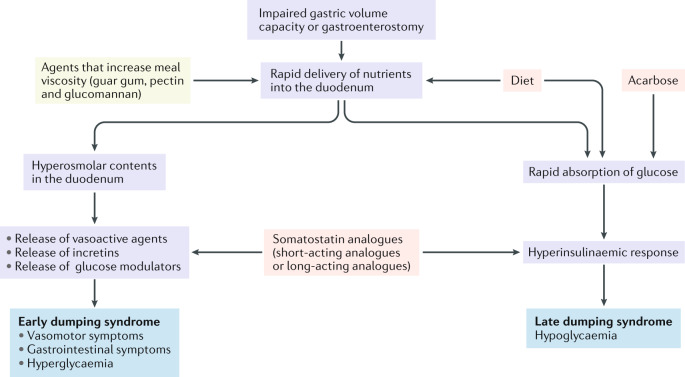
Pathophysiology and therapeutic targets in dumping syndrome. The pathophysiological flow chart of dumping syndromes is presented in purple, with the main features of early and late dumping syndromes presented in blue. Therapeutic agents that increase meal viscosity (such as guar gum, pectin and glucomannan) have no clear evidence of efficacy (yellow). By contrast, endorsed evidence of efficacy is available for the use of diet modifications, acarbose and somatostatin analogues (pink).

On the basis of the statements that generated consensus, a number of recommendations can be made for managing patients with dumping syndrome, which are summarized in Table 5. There is good agreement on definition, symptom pattern and underlying pathophysiological mechanisms. The pathophysiological concepts of dumping syndrome are summarized in Fig. 1. However, the Delphi process also identified several areas of uncertainty, which require further research.

**Table 5:** Recommendations from the Delphi consensus on dumping syndrome

Recommendations	Based on statements	Grading level
Dumping syndrome is a complication of oesophageal or gastric surgery that can comprise both early and late dumping syndrome symptoms	1–6	Grade B
Early dumping syndrome is the typical and most frequent manifestation of dumping syndrome and can occur in isolation or in association with late symptoms	4–6	Grades A and B
Dumping syndrome affects quality of life and can be associated with weight loss	7 and 8	Grade B
Symptoms of early dumping syndrome are driven by rapid delivery of nutrients to the small bowel, which triggers release of several gastrointestinal hormones, including vasoactive agents, incretins and glucose modulators	9–13	Grade B
Hypoglycaemia is the main symptom of late dumping syndrome, and is driven by a hyperinsulinaemic response and GLP1 release	14 and 15	Grades A and B
Dumping syndrome can contribute to weight loss after bariatric surgery	16 and 17	Grade B
Dumping syndrome should be suspected based on the clinical history, but currently available dumping questionnaires have no proven diagnostic value	19–26	Grades B and C
Spontaneous hypoglycaemia below 2.8 mmol/l (50 mg/dl) is suggestive of late dumping syndrome	27	Grade B
A modified oral glucose tolerance test is a useful diagnostic test for dumping syndrome. The test is considered positive for early dumping syndrome in case of an early (30 min) increase in haematocrit >3% or in pulse rate >10 bpm. The test is considered positive for late dumping syndrome in case of late (60–180 min after ingestion) hypoglycaemia (<50 mg/dl)	33–39	Grades B and C
The value of continuous glucose monitoring for diagnosing dumping syndrome has not been established	30 and 31	Grade C
Mixed meal tests are not considered superior to the modified glucose tolerance test, and gastric emptying tests have no established value in diagnosing dumping syndrome	40–42	Grades B and C
Dietary intervention, with elimination of rapidly absorbable carbohydrates, is the first-line treatment approach for dumping syndrome. Patients are also advised to consume high fibre and protein-rich foods, eaten slowly and chewed well	43–47	Grades B and C
Agents that increase meal viscosity have no established value in the management of dumping syndrome	48	Grade B
Acarbose is effective for the treatment of dumping syndrome symptoms, especially symptoms of late dumping syndrome	50 and 51	Grade B
Diazoxide has no established value for the treatment of dumping syndrome	52	Grade C
Somatostatin analogues are effective for the treatment of dumping syndrome. The short-acting analogues have greater efficacy but require multiple injections	53–56	Grade B
Continuous enteral or gastric feeding has no established value for the treatment of dumping syndrome	57 and 58	Grade C
Surgical interventions (or re-interventions) for dumping syndrome have uncertain outcomes and the optimal procedure is not established	59–62	Grades B and C

GLP1, glucagon-like peptide 1.

Dumping syndrome might contribute to the weight loss that occurs after bariatric surgery (statements 16 and 17). However, there is some controversy about whether symptoms of dumping syndrome after bariatric surgery can be useful for the patient’s awareness of unwanted food effects and hence contribute to the weight loss (statement 17)^11,55,60^. Furthermore, the literature shows no evidence that dumping syndrome improves or might contribute favourably to the weight loss after bariatric surgery (statement 17)^11,55,60^. Moreover, dumping syndrome impairs the quality of life of patients so it should be considered a deleterious complication in these patients. Nevertheless, additional prospective studies are warranted to further clarify this issue. The nature of the putative entity of ‘idiopathic dumping syndrome’ in patients who did not undergo surgery, which is characterized by rapid gastric emptying and associated symptoms, also lacks consensus (statement 18).

In terms of diagnosis, the available dumping syndrome severity questionnaires are not considered reliable enough to aid in the diagnostic process (statements 21–27). Furthermore, all of these questionnaires are thought to lack sensitivity to treatment interventions, indicating the need for a specific patient-reported outcome questionnaire to be developed for dumping syndrome. There is agreement that the modified OGTT is the preferred diagnostic method (statement 33), and diagnostic parameters for early and late dumping syndrome are well established (statements 34–36). The same glycaemia level cut-off (50 mg/dl) is proposed for spontaneous glycaemia levels and for late hypoglycaemia during the modified OGTT for supporting the diagnosis of dumping syndrome (statements 28, 29, 35 and 36). However, the reproducibility and sensitivity of the modified OGTT are not well established (statements 37–39). The value of continuous glucose monitoring in the diagnosis and management of dumping syndrome needs further research (statements 29, 31 and 32) and the mixed meal test is not considered a standard for diagnosis (statement 40). No diagnostic value is attributed to gastric emptying tests (statements 41 and 42).

There is agreement that a dietary approach, focusing on low-volume meals with elimination of rapidly absorbable carbohydrates, protein-rich and high-fibre foods and delay of fluid intake, is the preferred initial approach for the treatment of dumping syndrome (statements 43–47). The pathophysiological basis for dumping syndrome treatments is summarized in Fig. 1. In patients who do not respond to diet modifications, pharmacotherapy is advocated, and the use of acarbose is supported (statements 49 and 50), especially for late dumping syndrome; however, the effects of acarbose in early dumping syndrome are unclear (statement 51). The Delphi panel did not support the use of agents that increase meal viscosity or diazoxide (statements 48 and 52). In patients who do not respond to diet modifications or acarbose, somatostatin analogues are supported for their ability to control symptoms of both early and late dumping syndrome (statement 53). Short-acting analogues are considered superior to long-acting forms, but the need for repeated injections with short-acting agents is a limiting factor (statements 54–56). In patients who do not respond to treatment, the value of continuous enteral feeding and especially of surgical re-intervention and pancreatic resection is uncertain (statements 56–58 and 60–62) and a conservative, non-surgical approach is recommended (statement 59). A diagnostic and therapeutic algorithm based on the Delphi process is shown in Box 3.

Box 3 Diagnostic and treatment algorithm for dumping syndrome

- The presence of symptoms suggestive of early or late dumping syndrome in a patient who has undergone oesophageal or gastric surgery should raise clinical suspicion. Patients often mention the need to lie down after meals due to profound weakness.
- Standard diagnostic evaluation (using endoscopy and imaging, for instance) might be necessary to exclude other reasons for the symptoms (such as postoperative strictures, adhesions and insulinoma).
- The modified oral glucose tolerance test is the preferred diagnostic method to confirm the diagnosis of dumping syndrome, and diagnostic parameters for early dumping syndrome are well established: an increase in haematocrit >3% at 30 min or an increase in pulse rate >10 bpm after 30 min. Late hypoglycaemia is another indicator of dumping syndrome; there is agreement on a cut-off of 50 mg/dl and not of 60 mg/dl.
- Similarly, there is no consensus on the nadir spontaneous glycaemia level that supports the diagnosis of dumping syndrome.
- Dietary measures, focusing on low-volume meals with elimination of rapidly absorbable carbohydrates and delay of fluid intake, are the preferred initial approach.
- In patients who do not respond to diet modification, the use of acarbose is recommended, especially for late dumping syndrome, but with an unclear effect on early dumping syndrome.
- In patients who do not respond to diet and/or acarbose, somatostatin analogues can control symptoms of both early and late dumping syndrome. It is unclear whether short-acting analogues are superior to long-acting formulations.
- In patients who do not respond to treatments, the value of continuous enteral feeding and especially of surgical re-intervention and pancreatic resection is uncertain and a conservative approach is recommended.

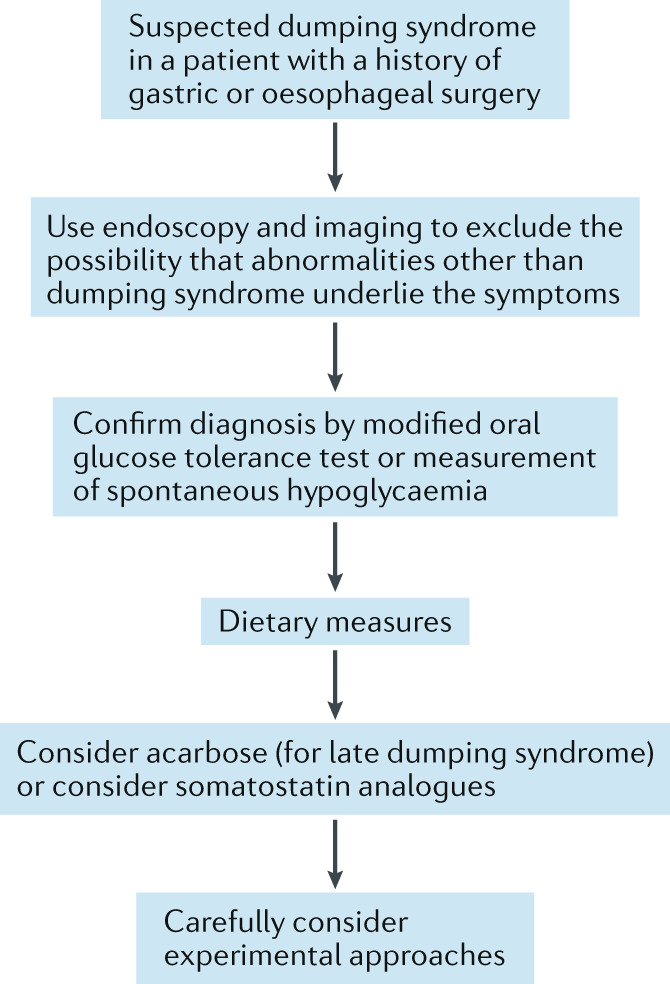

## Conclusion

Dumping syndrome is a prevalent but probably under-recognized complication of oesophageal and gastric surgery, including bariatric interventions. To date, there are no established guidelines on the diagnosis and management of dumping syndrome, and hence we organized a Delphi consensus process to establish the current state of knowledge, to provide guidance to clinicians and identify areas requiring future research.

The Consensus Group reached consensus on several aspects, including the definition, symptom pattern and presumed underlying pathophysiology. Clinical awareness and modified OGTT are the key methods for making a diagnosis of dumping syndrome. In addition to dietary measures, acarbose and somatostatin analogues are well accepted treatment modalities. The consensus process also identified areas in need of further research, such as the development and evaluation of diagnostic and outcome questionnaires, agreement of threshold glycaemia levels for reliable diagnosis, evaluation of the therapeutic efficacy of acarbose for symptoms of early dumping syndrome and assessment of the relative efficacy of short-acting versus long-acting somatostatin analogues. The role of mixed meal tests, gastric emptying tests, continuous enteral nutrition and surgical interventions for dumping syndrome also need further evaluation.
